# A comparison of the severity of tricuspid valve regurgitation between echocardiographic and cardiac magnetic resonance criteria in adults with congenital heart disease

**DOI:** 10.3389/fcvm.2026.1708683

**Published:** 2026-05-21

**Authors:** Chomchanok Tantasutthanon, Chanankarn Kamsorn, Tananya Lueangklanlayanakhun, Watcharee Prasertkulchai, Varinsawat Prakongwong, Tarinee Tangcharoen

**Affiliations:** 1 Department of Internal Medicine, Surat Thani Hospital, Surat Thani, Thailand; 2 Department of Internal Medicine, Mahidol University Ramathibodi Hospital, Bangkok, Thailand

**Keywords:** adult congenital heart disease, atrial septal defect, cardiac magnetic resonance (CMR), Ebstein’s anomaly, echocardiography criteria, repaired Tetralogy of Fallot, tricuspid regurgitation (TR)

## Abstract

**Purpose:**

This study aims to assess the prevalence of different degrees of tricuspid regurgitation (TR) severity using cardiac magnetic resonance (CMR) and echocardiographic criteria and to examine its effect on right ventricular size and function in patients with adult congenital heart disease (ACHD).

**Methods and results:**

CMR and 2D echocardiographic data from patients with Ebstein's anomaly, atrial septal defects, and repaired Tetralogy of Fallot who were followed up at a university hospital from 2011 to 2023 were retrospectively reviewed. Patients were excluded if the interval between CMR and echocardiography exceeded 6 months or if an arrhythmia occurred during scanning. TR severity was graded using qualitative and semi-quantitative parameters. A total of 115 patients were included. The median interval between the echocardiography and CMR was 42 days. Of 115 patients, 28 patients (24.3%) had discordant TR severity between echocardiography and CMR TR volume, and 25 patients (21.7%) had discordant TR severity between echocardiography and CMR TR fraction. The number of patients with moderate or severe TR was lower when the CMR criteria were applied. The highest proportion of varying TR severity was observed in patients with Ebstein's anomaly (72.7%). The right ventricular end-diastolic volume index did not differ statistically between patients with non-severe TR, as determined by CMR and echocardiographic criteria.

**Conclusions:**

In a specific group of ACHD patients, 25% showed discordant TR severity, with the highest prevalence in those with Ebstein's anomaly. Application of CMR criteria was found to lower the overall TR severity.

## Introduction

Tricuspid regurgitation (TR) is common in the adult population and is strongly associated with adult congenital heart disease (ACHD) (1). In adult congenital heart disease, tricuspid regurgitation can be either primary TR, such as in Ebstein's anomaly, or secondary TR due to right ventricular dilatation in patients with a cardiac shunt or pulmonic valve regurgitation (2). Increasing TR severity adversely affects right ventricular function and is associated with higher mortality; accordingly, surgical intervention is recommended for patients with ACHD and severe TR (3, 4). Accurate assessment of TR severity is therefore crucial. Although the American Heart Association/American College of Cardiology (AHA/ACC) and the European Society of Cardiology (ESC) guidelines for the management of ACHD patients recommend CMR for cardiac anatomical and functional evaluation, CMR has not played a significant role in valvular assessment, apart from pulmonic valve regurgitation, to the same extent as echocardiography. The American Society of Echocardiography (ASE) has recently proposed criteria for assessing TR severity using echocardiography and cardiac magnetic resonance (CMR) (5). The echocardiographic criteria comprise qualitative, semi-quantitative, and quantitative parameters. For CMR, TR severity is graded using either tricuspid regurgitation volume or tricuspid valve regurgitation fraction. However, the ASE guidelines also state that the CMR criteria are based on those established for mitral regurgitation (5).

A recent study compared the accuracy of TR severity assessment between echocardiography and CMR (6). Using the 2017 American Society of Echocardiography multiparametric criteria, only 65% of patients agreed with CMR-derived TR severity. However, agreement with CMR using a hierarchical approach was significantly higher than that with the 2017 ASE criteria. Nevertheless, this study was conducted in non-ACHD patients, and data comparing TR severity criteria between echocardiography and CMR remain limited. Since CMR is the gold standard for evaluating cardiac structure and function in ACHD patients, we aim to assess the prevalence of discordance between CMR and 2D echocardiographic TR severity using qualitative and semi-quantitative criteria in this patient group. We also aim to assess the impact of different criteria on cardiac function and TR progression.

## Materials and methods

### Study design

This retrospective, cross-sectional study was conducted at a single university hospital. The electronic medical records of adult patients with congenital heart disease who underwent CMR scanning between 2011 and 2023 were reviewed. Due to the heterogeneity of adult congenital heart disease, we decided to select patients with Ebstein's anomaly, atrial septal defects (ASD), or repaired Tetralogy of Fallot (rTOF), as tricuspid regurgitation is common in these three conditions. The inclusion criteria were (1) age >18 years old; (2) both transthoracic echocardiography (TTE) and CMR performed at our institute; and (3) an interval of less than 6 months between TTE and CMR. Exclusion criteria were (1) atrial fibrillation or cardiac arrhythmia during CMR scanning; (2) diuretic adjustment during the TTE–CMR interval; (3) inadequate imaging quality; and (4) presence of a prosthetic tricuspid valve.

Baseline characteristics and details of congenital heart disease, including cardiac surgery type, echocardiographic parameters, and cardiac magnetic resonance data, were retrieved from the electronic medical records. The study was approved by the Institutional Review Board and Ethics Committee of Ramathibodi Hospital (MURA 2023/432).

### Tricuspid regurgitation severity evaluation

The severity of tricuspid regurgitation was graded as mild, moderate, or severe based on qualitative and semi-quantitative parameters obtained from 2D transthoracic echocardiography, including continuous-wave density (CWD), vena contracta width (VCW), PISA radius, and hepatic vein flow pattern. Severe TR was defined by the presence of a dense, triangular CWD, VCW > 0.7 cm, PISA radius >0.9 cm, and systolic reversal of hepatic vein flow. Mild TR was defined as an incomplete or faint CWD, VCW <0.3 cm, PISA radius <0.4 cm, and systolic-dominant hepatic vein flow. Tricuspid regurgitation was graded as mild or severe TR when >50% of the corresponding criteria were met. TR was graded as moderate if fewer than 50% of the criteria parameters were met. According to the 2017 guidelines, quantitative measurements should be obtained in patients with equivocal TR severity after qualitative and semi-qualitative assessment. However, due to the retrospective design of the study, quantitative parameters were available for only 45.83% of patients with moderate TR, and assessment was limited to EROA. We therefore decided to grade TR severity based solely on qualitative and semi-qualitative parameters.

For cardiac magnetic resonance imaging, TR severity grading was assessed using either TR volume, calculated by subtracting pulmonary artery forward flow, measured by phase-contrast imaging, from the right ventricular systolic volume (RVSV), or TR fraction, calculated by dividing TR volume by RVSV. Because RVSV can be affected by either the volume of pulmonic regurgitation in rTOF patients or by significant left-to-right shunting in ASD patients, we decided to evaluate both TR volume and TR fraction grading and compare them with echocardiographic severity grading. Using TR volume, TR was graded as mild if the TR volume was <30 and severe if the TR volume was ≥45 mL. Using the TR fraction, TR was graded as mild if the TRF was <30% and severe if the TRF was ≥50%. This grading was based on a previous study demonstrating that these threshold values were associated with adverse outcomes in patients with tricuspid regurgitation (7). All CMR data analyses were performed by a cardiologist with more than 10 years of experience in CMR.

Right ventricular end-diastolic volume (RVEDV) and right ventricular ejection fraction (RVEF) were calculated from the CMR data. In patients with ASD and PR, the volumes attributable to ASD and PR were subtracted from the RVEDV to assess the true effect of TR on the RVEDV. In patients with Ebstein's anomaly, the atrialized RV was excluded from the RVEDV. RVEDV was indexed to body surface area. CMR sequence acquisition and post-processing analysis of right ventricular volume, function, and flow were performed as recommended by recent CMR guidelines (8, 9).

### Outcomes

The primary outcome was the prevalence of discordant TR severity between the two imaging criteria and of patients with different congenital diagnoses. Agreement between TR severity grades obtained by different imaging criteria was also evaluated. We analyzed the number of discordances and agreements for at least a one-grade difference in severity and between non-severe and severe TR criteria. The secondary outcome was the impact of TR severity on right ventricular size and function.

### Statistical analysis

Continuous variables were presented as the mean ± SD or as the median with the 25th and 75th quartiles and compared using a Student’s *t*-test for normally distributed data or a Mann–Whitney *U* test for non-normally distributed data. Categorical variables were described as numbers and percentages and compared using the chi-square test. Agreement between TR severity graded by echocardiography and CMR criteria was assessed using the weighted kappa test. The calculated kappa coefficients were interpreted as follows: 0–0.2 (low), 0.2–0.4 (fair), 0.4–0.6 (moderate), 0.6–0.8 (substantial), and >0.8 (excellent). *P* values <0.05 were considered statistically significant. All statistical analyses were performed using SPSS.

## Results

Between 2011 and 2023, 728 adult patients with congenital heart disease underwent CMR scanning at our institute. After excluding patients with an echocardiography–CMR interval greater than 180 days, those with arrhythmia during CMR, and those who received diuretic dose adjustment during the echocardiography–CMR interval, 115 patients were included in the study [70 women (60.9%), mean age 36 ± 15 years, median age 32 years]. Seventy-three patients (63.5%) were in NYHA class I, whereas 37 (32.2%) and 5 (4.3%) patients were in NYHA classes II and III, respectively. There were 12 patients with Ebstein's anomaly, 34 patients with ASD, and 69 patients with rTOF. Among patients with rTOF, 32 (46.38%) had significant PR (PR fraction >20%). Among those with ASD, 26 (76.47%) had a significant left-to-right shunt (Qp:Qs > 1.5). The median time interval between echocardiography and CMR was 42 days (Q1–Q3: 9–97 days).

### Different TR severity criteria across imaging modalities

**Figure 1:**
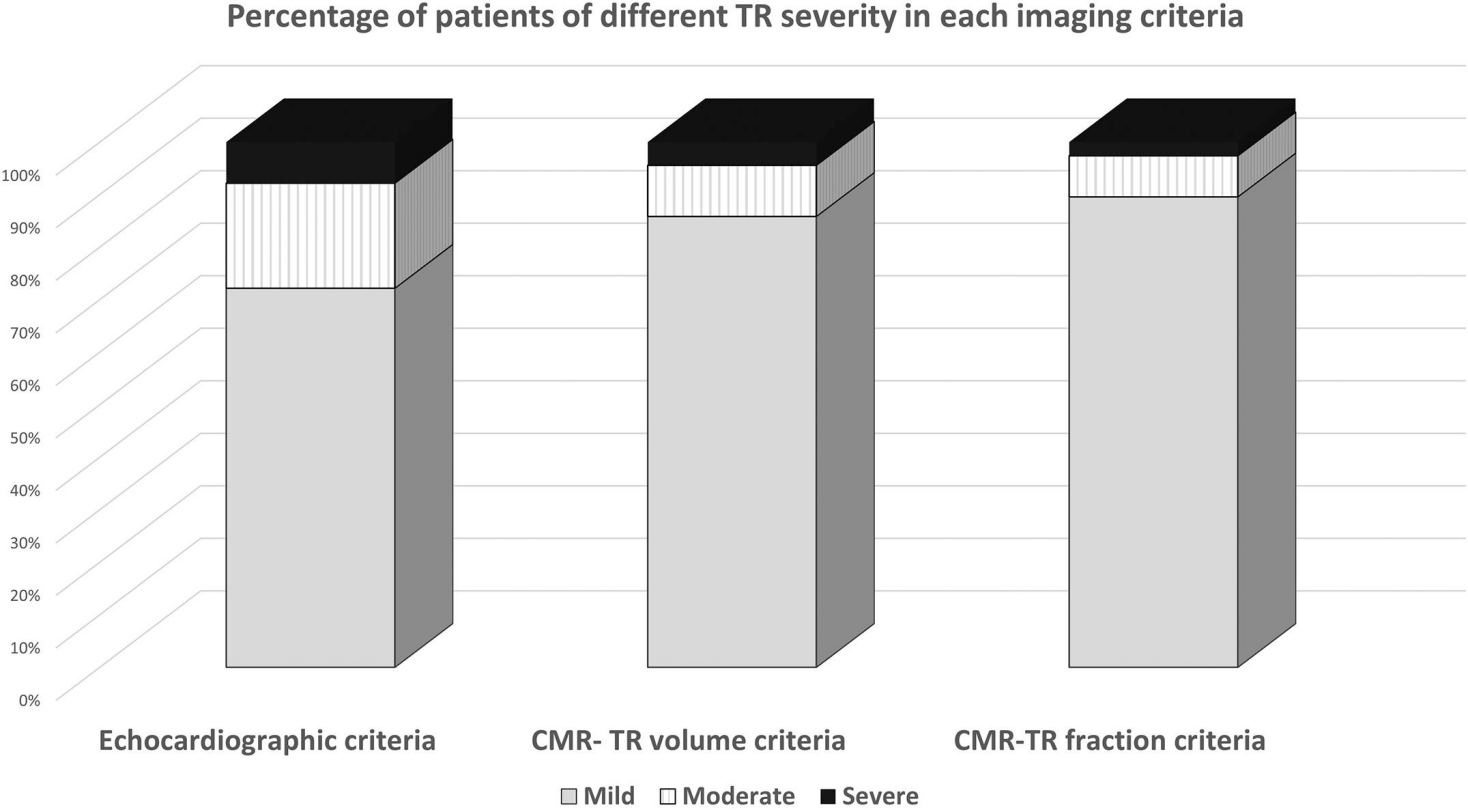
Number of patients with TR grading by echocardiography, CMR TR volume, and CMR TR fraction criteria.

By echocardiographic criteria, 83 patients (72.2%) had mild TR, 23 (20%) had moderate TR, and 9 (7.8%) had severe TR. In contrast, using CMR TR volume and TR fraction criteria, 97 (84.3%) and 103 (89.6%) patients had mild TR; 13 (11.3%) and 9 (7.8%) patients had moderate TR; and 5 (4.3%) and 3 (2.6%) patients had severe TR, respectively. Figure 1 demonstrates the number of patients with TR grading by echocardiography, CMR TR volume, and CMR TR fraction criteria. Compared with echocardiographic criteria, the number of patients with mild and moderate TR was higher when using CMR, by both volume and fraction criteria.

### Discordant TR severity and agreement between echocardiographic and CMR-derived criteria

There were 28 out of 115 patients (24.3%) who had at least one grade of TR severity discordance between echocardiography and CMR TR volume and 25 out of 115 patients (21.7%) between echocardiography and CMR TR fraction. The majority of patients with different TR severity were in the moderate TR group by echocardiography. Of the 23 patients with moderate TR by echocardiography, 19 (82.6%) were classified as having mild TR by CMR TR volume criteria. In contrast, 78 of 93 patients (83.9%) with mild TR by echocardiography remained classified as mild by CMR. Agreement was fair for both echocardiography–CMR TR volume criteria and echocardiography–CMR TR fraction criteria, with kappa values of 0.333 (95% CI, 0.315−0.351; *p* < 0.001) and 0.351 (95% CI, 0.335−0.367; *p* < 0.001), respectively. Agreement between CMR TR volume criteria and CMR TR fraction criteria was moderate (kappa=0.48; 95% CI, 0.458–0.502; *p* < 0.001).

When considering discordance in non-severe and severe TR, the number of patients with discordant severity criteria decreased to 4 of 115 (3.48%) patients for echocardiography–CMR TR volume criteria and 6 of 115 (5.22%) for echocardiography–CMR TR fraction criteria (Table 1). This resulted in substantial agreement between echocardiography and CMR TR volume criteria, with a kappa of 0.732 (95% CI, 0.710–0.754; *p* < 0.001), and moderate agreement between echocardiography and CMR TR fraction criteria, with a kappa of 0.562 (95% CI, 0.535–0.589; *p* < 0.001).

**Table 1:** Number of patients with each TR severity level, as determined by echocardiography and various CMR criteria.

CMR criteria	Degree of severity	Echocardiography criteria	
		Non-severe	Severe
CMR TR volume criteria	Non-severe	106	**4**
	Severe	0	5
CMR TR fraction criteria	Non-severe	106	**6**
	Severe	0	3

Bold numbers indicate patients with discordant criteria.

### Different TR severity between different congenital heart diseases

**Figure 2:**
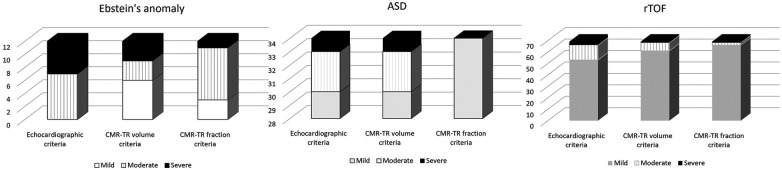
Number of patients with each TR severity grade according to different imaging criteria for each congenital heart disease.

Figure 2 shows the number of patients with each TR severity grade according to different imaging criteria for each congenital heart disease. Patients with Ebstein's anomaly showed the highest discordance in TR severity compared with those with ASD and rTOF. Eight out of 12 patients (66.67%) with Ebstein's anomaly had at least a one-grade discordance in TR severity between echocardiography and CMR TR volume criteria, whereas 4 of 34 (11.76%) patients with ASD and 16 of 69 (23.19%) patients with rTOF had at least a one-grade discordance. This resulted in poor kappa agreement between echocardiography and CMR-derived criteria in patients with Ebstein's anomaly. Table 2 demonstrates the kappa agreement for each congenital heart disease. Agreement between echocardiographic and CMR TR fraction criteria was not assessed in ASD patients because all patients had mild TR by CMR TR fraction, so other severity grades could not be analyzed.

**Table 2:** Kappa agreement between echocardiographic and CMR-derived TR severity criteria for each congenital heart disease.

Disease category	Comparison between imaging modalities	Kappa	95% CI	*p*
Overall	Echo vs. CMR TR volume criteria	0.333	0.315–0.351	<0.001
	Echo vs. CMR TR fraction criteria	0.351	0.335–0.367	<0.001
	CMR TR volume vs. TR fraction criteria	0.480	0.458–0.502	<0.001
Ebstein's anomaly	Echo vs. CMR TR volume criteria	0.111	0.082–0.140	0.408
	Echo vs. CMR TR fraction criteria	−0.012	−0.05 to 0.02	0.939
	CMR TR volume vs. TR fraction criteria	0.394	0.361–0.427	0.018
ASD	Echo vs. CMR TR volume criteria	0.447	0.401–0.493	0.005
	Echo vs. CMR TR volume criteria	N/A	N/A	N/A
	CMR TR volume vs. TR fraction criteria	N/A	N/A	N/A
rTOF	Echo vs. CMR TR volume criteria	0.230	0.206–0.254	0.018
	Echo vs. CMR TR volume criteria	0.217	0.195–0.239	0.001
	CMR TR volume vs. TR fraction criteria	0.521	0.485–0.557	<0.001

ASD, atrial septal defect; rTOF, repaired Tetralogy of Fallot.

### Effect of different TR severity on right ventricular size and function

Due to the small number of patients with severe TR, comparisons of right ventricular end-diastolic volume index (RVEDVi) and RVEF across different imaging criteria were limited to patients with non-severe TR (Table 3). RVEDVi and RVEF were not significantly different between echocardiographic and CMR-derived criteria (*p* = 0.834 and 0.90 for RVEDVi and RVEF in echocardiographic vs. CMR TR volume criteria and *p* = 0.79 and 0.609 for RVEDVi and RVEF in echocardiographic vs. CMR TR fraction criteria, respectively).

**Table 3:** Right ventricular size and function in non-severe TR, as defined by echocardiographic and CMR-derived criteria.

Right ventricular parameters	Non-severe TR		
	Echocardiography criteria	CMR TR volume criteria	CMR TR fraction criteria
RVEDVi (mL/m^2^)	**94.51** (Q1–Q3: 78.81–114.84)	**95.58** (Q1–Q3: 80.09–116.54)	**96.14** (Q1–Q3: 80.47–118.13)
RVEF (%)	53.80 ± 9.26	53.65 ± 9.62	53.51 ± 9.68

RVEDVi, right ventricular end-diastolic volume index; RVEF, right ventricular ejection fraction; ASD, atrial septal defect; rTOF, repaired Tetralogy of Fallot; Bold value, median value; Q1–Q3, 1st Quartile–3rd Quartile.

### Effect of significant PR and left-to-right shunt on agreement in TR severity

The degree of left-to-right shunt and pulmonic regurgitation did not significantly affect the discordance between echocardiographic and CMR criteria; similarly, the interval between echocardiography and CMR, in addition to RVEDVi and RVEF, showed no significant association with discordance, as demonstrated in Table 4.

**Table 4:** Regression analysis of predictors of discordance in agreement between echocardiographic and CMR-derived criteria.

Parameters	Sig	Exp (*B*)	95% CI for Exp (*B*)	
			Lower	Upper
Duration between echocardiography and CMR (days)	0.978	1	0.992	1.007
RVEDVi (mL/m^2^)	0.994	1	0.995	1.005
RVEF (%)	0.595	0.986	0.936	1.038
Qp:Qs > 1.5	0.371	0.558	0.156	2.003
Significant pulmonic regurgitation	0.948	0.968	0.359	2.612

RVEDVi, right ventricular end-diastolic volume index; RVEF, right ventricular ejection fraction.

### Clinical follow-up

Two patients with Ebstein's anomaly underwent tricuspid valve replacement: one was diagnosed with severe TR by both echocardiographic and CMR criteria, while the other was diagnosed with moderate TR. Among patients with ASD, 21 underwent either surgical or transcatheter ASD closure. One patient with severe TR by echocardiographic and CMR criteria underwent surgical ASD closure and tricuspid valve repair. Among patients with rTOF, 29 underwent either surgical or transcatheter pulmonic valve replacement. None underwent tricuspid valve intervention. The effects of different degrees of TR severity on TR progression and RV function could not be assessed due to the small number of patients with echocardiographic follow-up (27 of 63 patients who did not receive any intervention).

## Discussion

Our study found that, using qualitative and semi-quantitative echocardiographic criteria, up to 25% of adult patients with congenital heart disease exhibited at least a one-grade difference in TR severity between echocardiographic and CMR-derived TR volume and TR fraction criteria. The number of patients with discordant severity reduced to 4% when only severe and non-severe criteria were considered. Patients with Ebstein's anomaly had the highest prevalence of discordant grading (72.3%). Overall, TR severity was lower when assessed using the CMR criteria. Agreement between echocardiographic and CMR-derived assessments of TR severity was moderate. RVEDVi and RVEF did not differ significantly between patients with non-severe TR as defined by echocardiography and CMR.

Our findings are consistent with those of Zhan et al., who reported that only 65% of TR severity assessments based on the 2017 ASE guidelines agreed with CMR-derived TR severity. Although current guidelines for TR severity evaluation include qualitative, semi-qualitative, and quantitative 2D echocardiographic parameters, none are 100% specific for severe or mild TR. Therefore, the guidelines recommend a multiparametric approach. Zhan et al. proposed a hierarchical severity approach with multiple echocardiographic parameters, including quantitative regurgitant volume. This new approach improves the accuracy of echocardiographic assessment of TR severity. However, this new method has not been validated in ACHD patients, and some parameters, such as RV size, may not be useful for TR severity assessment in these patients, especially in those with pre-tricuspid shunts or significant PR, where the RV is already enlarged. Some echocardiographic parameters also have limitations in ACHD. The continuous-wave Doppler density and hepatic vein reversal flow may be observed in patients with mild TR but with severe right atrial pressure elevation or reduced right atrial and right ventricular compliance. Right ventricular diastolic dysfunction, which results in increased right atrial filling pressure and a right ventricular restrictive filling pattern, is not uncommon in patients with congenital heart disease (10, 11). This could affect hepatic vein flow in ACHD patients. Our study showed that patients with Ebstein's anomaly demonstrated the greatest difference in TR severity between echocardiographic and CMR-derived criteria. Echocardiographic quantification of TR in Ebstein's anomaly is challenging. The regurgitant jet can be directed inferiorly, resulting in suboptimal visualization in an apical four-chamber view (12). Vena contracta width cannot be accurately assessed in the presence of multiple TR jets, while the severely dilated right atrium and the atrialized right ventricle can affect right atrial compliance and hepatic venous flow (13). In our study, two patients with Ebstein's anomaly had severe TR by echocardiography, with hepatic reversal flow. However, CMR demonstrated TR volumes of 35.5 and 35.3 mL/beat, corresponding to moderate TR by CMR TR volume criteria. Although the 3D vena contracta area has been suggested as a better parameter than 2D measurements for primary TR (14), no studies have yet been conducted in ACHD populations.

Although agreement between echocardiographic and CMR-derived criteria in our study was moderate, it improved to substantial when considering only severe and non-severe TR. Identifying patients with severe TR is relevant because current clinical practice guidelines for adult congenital heart disease recommend tricuspid valve intervention in patients with severe tricuspid regurgitation. However, in patients with moderate tricuspid regurgitation, tricuspid valve intervention should be considered under specific circumstances. According to the 2025 ACC/AHA guidelines for the management of adult congenital heart disease, pulmonic valve replacement may be considered (class IIb) in rTOF patients who have moderate pulmonic regurgitation and moderate or greater tricuspid regurgitation associated with RV dilatation, with the aim of preventing progression of tricuspid regurgitation (15). In patients with Ebstein's anomaly, tricuspid valve repair is recommended in moderate TR in the presence of heart failure symptoms, declining exercise capacity, or progressive RV dysfunction. In these two clinical circumstances, accurate identification of patients with moderate TR is critical for clinical management. Our study demonstrated that the majority of discordance in TR severity occurred in patients classified as having moderate TR by echocardiography, with CMR assigning lower TR severity in the majority of cases. Nevertheless, our study lacked clinical outcome data; therefore, the clinical significance of lowering TR severity in CMR-derived criteria remains uncertain. Its clinical significance needs to be studied in a larger patient cohort.

The cutoff values for CMR-derived TR severity that we used in our study have already been evaluated by Zahn et al. (7). They showed that using CMR TR volume ≥45 mL and CMR TR fraction ≥50% can identify patients in the highest mortality strata, with both TR volume and TR fraction associated with increased mortality after adjusting for imaging and clinical covariates. However, it should be noted that the patients included in that study did not have congenital heart disease. The evaluation of TR severity in patients with congenital heart disease, as recommended in the ACHD guidelines, relies mainly on echocardiographic criteria. Our study highlights the need for a larger prospective study comparing the echocardiographic hierarchical approach with CMR TR volume and fraction criteria in this patient group. Future cohort studies to identify the CMR cutoff values for clinical outcomes are also needed.

Recently, several publications have argued that the three-grade scheme recommended by the ASE should be re-stratified into a four- or five-grade scheme using the quantitative echocardiographic parameters, including EROA and PISA, to provide better prognostic information (16–18). Using the four- or five-grade scheme, patients with moderate TR are graded as mild-to-moderate or moderate-to-severe. Because quantitative parameters were available in only 45.83% of our patients with moderate TR, and assessment was limited to EROA, it should be noted that the discordance between echocardiographic and CMR grading in our study applies to patients with qualitative and semi-quantitative echocardiographic data, rather than those undergoing full quantitative assessment.

### Limitations

Our study has several limitations. First, it was a single-center, retrospective study with a small sample size. Therefore, multicenter prospective studies with larger sample sizes are required. Second, due to the retrospective nature of the study, not all patients underwent quantitative echocardiographic evaluation of TR. Therefore, our data consisted solely of qualitative and semi-qualitative parameters and did not meet the hierarchical approach recommended by the 2017 ASE guidelines. Third, although we evaluated the impact of TR severity, defined by different criteria, on right ventricular size and function, we did not assess its effect on right ventricular remodeling after tricuspid valve surgery. Fourth, our data did not include 3D echocardiography, which is essential in non-ACHD patients. Therefore, the discordance between echocardiographic and CMR criteria in our study applies only to patients who underwent qualitative or semi-quantitative assessment. Fifth, although CMR data were analyzed by a cardiologist with more than 10 years of CMR experience, inter- and intra-observer variability was not assessed. Finally, it is worth noting that our study was an imaging-based study without clinical outcome data; therefore, the clinical significance of discordance between echocardiographic and CMR-derived TR grading remains uncertain. Its clinical significance needs to be studied in a larger patient cohort.

## Conclusions

Using current cardiac magnetic resonance and qualitative/semi-quantitative echocardiographic criteria for TR severity in a specific group of ACHD patients, discordance was observed in 25% of cases, with the highest prevalence in patients with Ebstein's anomaly. CMR criteria lower the overall TR severity. However, the effect of using different TR criteria on right ventricular size and function was not significantly different in patients with non-severe TR.

## Data Availability

The anonymous data supporting the conclusions of this article will be provided if requested to the authors.
